# A scalable corneal xenograft platform: simultaneous opportunities for tissue engineering and circular economic sustainability by repurposing slaughterhouse waste

**DOI:** 10.3389/fbioe.2023.1133122

**Published:** 2023-04-25

**Authors:** Xinyu Wang, Adeeba Shakeel, Ahmed E. Salih, Hema Vurivi, Sayel Daoud, Luca Desidery, Raheema L. Khan, Meklit G. Shibru, Zehara M. Ali, Haider Butt, Vincent Chan, Peter R. Corridon

**Affiliations:** ^1^ Biomedical Engineering and Healthcare Engineering Innovation Center, Khalifa University, Abu Dhabi, United Arab Emirates; ^2^ Department of Immunology and Physiology, College of Medicine and Health Sciences, Khalifa University of Science and Technology, Abu Dhabi, United Arab Emirates; ^3^ Department of Mechanical Engineering, College of Medicine and Health Sciences, Khalifa University of Science and Technology, Abu Dhabi, United Arab Emirates; ^4^ Center for Biotechnology, Khalifa University of Science and Technology, Abu Dhabi, United Arab Emirates; ^5^ Anatomical Pathology Laboratory, Cleveland Clinic Abu Dhabi, Abu Dhabi, United Arab Emirates; ^6^ Department of Civil Infrastructure and Environmental Engineering, College of Engineering, Khalifa University of Science and Technology, Abu Dhabi, United Arab Emirates

**Keywords:** decellularization, corneas, keratoprosthesis, xenograft, tissue engineering, circular economy

## Abstract

**Introduction:** Corneal disease is a leading cause of blindness globally that stems from various etiologies. High-throughput platforms that can generate substantial quantities of corneal grafts will be invaluable in addressing the existing global demand for keratoplasty. Slaughterhouses generate substantial quantities of underutilized biological waste that can be repurposed to reduce current environmentally unfriendly practices. Such efforts to support sustainability can simultaneously drive the development of bioartificial keratoprostheses.

**Methods:** Scores of discarded eyes from the prominent Arabian sheep breeds in our surrounding region of the United Arab Emirates (UAE) were repurposed to generate native and acellular corneal keratoprostheses. Acellular corneal scaffolds were created using a whole-eye immersion/agitation-based decellularization technique with a widely available, eco-friendly, and inexpensive 4% zwitterionic biosurfactant solution (Ecover, Malle, Belgium). Conventional approaches like DNA quantification, ECM fibril organization, scaffold dimensions, ocular transparency and transmittance, surface tension measurements, and Fourier-transform infrared (FTIR) spectroscopy were used to examine corneal scaffold composition.

**Results:** Using this high-throughput system, we effectively removed over 95% of the native DNA from native corneas while retaining the innate microarchitecture that supported substantial light transmission (over 70%) after reversing opacity, a well-established hallmark of decellularization and long-term native corneal storage, with glycerol. FTIR data revealed the absence of spectral peaks in the frequency range 2849 cm^−1^ to 3075 cm^−1^, indicating the effective removal of the residual biosurfactant post-decellularization. Surface tension studies confirmed the FTIR data by capturing the surfactant’s progressive and effectual removal through tension measurements ranging from approximately 35 mN/m for the 4% decellularizing agent to 70 mN/m for elutes highlighting the effective removal of the detergent.

**Discussion:** To our knowledge, this is the first dataset to be generated outlining a platform that can produce dozens of ovine acellular corneal scaffolds that effectively preserve ocular transparency, transmittance, and ECM components using an eco-friendly surfactant. Analogously, decellularization technologies can support corneal regeneration with attributes comparable to native xenografts. Thus, this study presents a simplified, inexpensive, and scalable high-throughput corneal xenograft platform to support tissue engineering, regenerative medicine, and circular economic sustainability.

## 1 Introduction

There are renewed interests in recycling unsuitable donor tissues (Bernard et al., 2015; Xie et al., 2021; Khan et al., 2023) and xenotransplantation (Wilson et al., 2013; Yoon et al., 2021; Fischer and Schnieke, 2022) to increase the supply of transplantable corneal grafts for keratoprosthesis. Such interests rely on preserving macroscopic tissue transparency, light transmittance, and vital corneal components, namely, the extracellular matrix (ECM) (Khan et al., 2023). In parallel, advances in tissue engineering also support the efforts to enhance biocompatibility and reduce the risk of zoonosis, and ultimately our ability to generate patient-specific grafts (Yoon et al., 2021). Combining these efforts could reverse the existing supply/demand discrepancy. Furthermore, xenografts generated from porcine and ovine options may generate enough transplantable grafts to address the current and growing needs worldwide. Corneal eye-bank tissues unsuitable for transplantation have been previously investigated for their potential to address problems associated with cadaveric donor shortages (Wilson et al., 2016). Another abundant source of corneal tissues is the slaughterhouse, which generates ample quantities of underutilized biological waste (Khan et al., 2023). As a result, procuring discarded eyes from abattoirs can simultaneously provide unique opportunities to produce bioartificial keratoprostheses and drive sustainable bioeconomic practices by repurposing this waste.

The literature presents a few novel approaches for generating corneal scaffolds that combine decellularization with other material fabrication techniques (Wang Xe et al., 2023). Thus, there is a need for further research in this area. Accordingly, this research highlights detailed approaches for creating a simplified, inexpensive, and scalable corneal xenograft platform. The platform uses discarded whole eyes from local abattoirs to create corneal grafts or scaffolds that can be used for various purposes, namely, *in vitro* and *in vivo* models that can ultimately generate patient-specific keratoprostheses, and provide a platform for pharmacological testing and cell and gene therapies that are poised to support bioartificial organ development (Kong and Mi, 2016; Ahearne et al., 2020a; Wang Xe et al., 2023). Our data extensively outlines ways to effectively generate scaffolds that preserve the appreciable levels of ocular transparency, transmittance, and ECM components for applicability as native ovine grafts (Niu et al., 2019), which can be genetically modified to reduce rejection and retrovirus transmission in the recipient (Song and Pan, 2019). Comparable characterizations are carried out for scaffolds created from decellularization, which produces extracellular matrix-based substrates with attributes similar to the native xenografts for driving corneal regeneration (Wilson et al., 2016; Polisetti et al., 2021). This process also sets the stage to develop associated artificial intelligence approaches that we can devise to can support xenograft characterizations (Shakeel and Corridon, 2022a; Corridon et al., 2022; Davidovic et al., 2022; Corridon, 2023a; Pantic et al., 2023a; Pantic et al., 2023b; Pantic et al., 2023c; Pantic et al., 2023d).

To the best of our knowledge, this is the first dataset to be generated in the United Arab Emirates (UAE) devised on repurposing slaughterhouse waste in a manner that supports circular economic practices. These efforts outline a fundamental step in developing a platform that can produce dozens of native ovine xenografts and acellular corneal scaffolds that effectively preserve ocular transparency, transmittance, and ECM components. It is well-established that decellularization technologies can support corneal regeneration with key attributes comparable to native xenografts (Shafiq et al., 2012; Ahearne et al., 2020b; Fernández-Pérez and Ahearne, 2020; Isidan et al., 2021). Even though decellularized scaffolds provide proper anatomical structures and cues for cell proliferation, differentiation, and organization into effectively functioning tissue (Moffat et al., 2022), there is a limited understanding of how we can manipulate the tissue microarchitecture to withstand *in vivo* environment (Polisetti et al., 2021). Several microscopic and macroscopic approaches have been applied to investigate these issues to provide an ideal setting for structural and functional evaluations, as well as recellularization techniques that can, in turn, support long-term transplantation (Isidan et al., 2021). In so doing, our approach can provide a means to generate further research. Thus, the novelty lies in this work’s ability to simultaneously provide opportunities for tissue engineering and circular economic sustainability by repurposing slaughterhouse waste and the simplicity with which a scalable experimental corneal xenograft platform can be created using governmental support.

## 2 Materials and methods

### 2.1 Study design

Discarded whole ovine eyeballs from the prominent Arabian sheep breeds [Najdi, Awassi (Nuaimi), and Orb] in our surrounding region of the UAE were repurposed to generate native and acellular corneal keratoprostheses. All sheep eyeballs were obtained from a local slaughterhouse in Abu Dhabi. DNA quantification, histological analyses, Fourier-transform infrared (FTIR) spectroscopy, surface tension, and optical transmittance experiments were performed using native or decellularized corneas to investigate their keratoprosthetic potential. These experiments were performed in accordance with the ARRIVE criteria and after approvals from the Automated Slaughterhouse of the Municipality of Abu Dhabi City and the Animal Research Oversight Committee at Khalifa University of Science and Technology (Abu Dhabi, United Arab Emirates). Further experimental details are presented in the subsequent sections.

### 2.2 Corneal extraction and decellularization

Cadaveric eyes were excised from the ocular globe, washed several times in saline placed in a sealed container with saline, and kept on ice in a cooler for subsequent transportation from the abattoir to the research facility. We then collected several batches of eyes and randomly selected eyes for the native and decellularization experimental groups. The native grafts were obtained by extracting corneas with the limbus intact and placing them as a group again in cold storage until further experimentation. The acellular corneal scaffolds were created using whole-eye post-immersion/agitation-based decellularization, in which 15–20 eyes were decellularized in a single chamber during this procedure.

**FIGURE 1:**
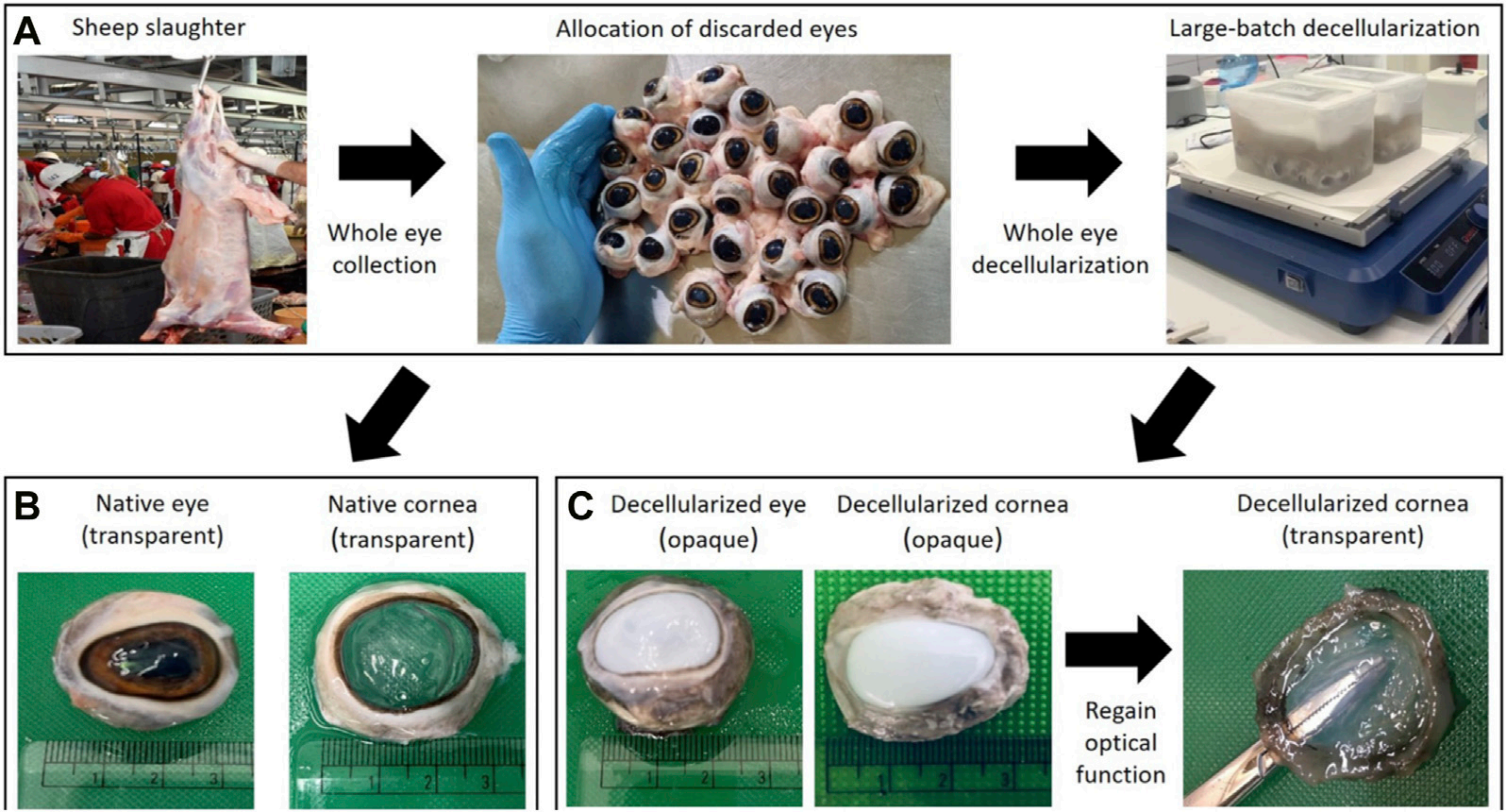
A schematic of the high-throughput system used to generate corneal tissues from slaughterhouse waste. Photographs in panel **(A)** illustrate the whole eye allocation and large-batch decellularization processes post-slaughter. Photographs in panel **(B)** highlight the retention of ocular transparency in native corneas using an index-matching agent (glycerol), while those in panel **(C)** display the reversal of ocular opacity in decellularized tissues using this clearing agent. Opacity is an established consequence of certain forms of corneal extraction and storage, and decellularization.

For decellularization, the isolated eyes (16 and 17 eyes in two containers separately) were immersed in 1 L of a 4% zwitterionic biosurfactant solution (Ecover, Malle, Belgium) that consisted of 15%–30% non-ionic surfactants, 5%–15% anionic surfactants, ethanol, sodium citrate, glycerin, trisodium ethylenediamine disuccinate, polypropylene terephthalate, and citric acid. Each chamber was set up on an Ohaus™ Analog Heavy Duty Shaker (Thermo Fischer Scientific, Waltham, MA, United States) and agitated for 7 days at a speed of 300 rpm. After this decellularization procedure, the eyes were removed from the detergent solution, and the decellularized corneas were extracted. These decellularized tissues were then subjected to an extensive washout process. For this process, the decellularized scaffolds were immersed in a similar volume of deionized water (1 L) and agitated again at 300 rpm for 3 days to remove the residual detergent from the tissues (Figure 1). During the first 2 days, the washout solution was changed 3X daily and replenished with fresh water, and then at the end of third day, the deionized water was changed for a final time. After which, the tissues were sectioned for further analysis, as presented below.

### 2.3 DNA quantification

DNA extraction was performed using QIAamp DNA Mini Kit #51306 (Qiagen, Germantown, MD, United States) according to manufacturer instructions and previously published protocol (Gerasimidis et al., 2016; Panek et al., 2018; Mousa et al., 2021; Kumar and Tejashree, 2022). Approximately 20 mg of tissue was cut into small pieces and subjected to overnight lysis. DNA was bound onto a silica membrane spin column, and further purification washes were done using wash buffers provided in the kit. Finally, DNA was eluted by using an elution buffer. The Nanodrop Spectrophotometer (ThermoFisher Scientific) was applied to assess the DNA quantity and purity. DNA quality was checked on 1% agarose gel, and the yield was recorded.

### 2.4 Histological assessments

A CX43 microscope (Olympus Corporation, Tokyo, Japan) equipped with 10X and 20X objectives was used to acquire brightfield micrographs of the various epithelial, Bowman’s, stromal, Descent’s, endothelial, and limbal layers. Corneas were fixed with 10% formalin for 1 h at 4°C. Specimens were rinsed in distilled H_2_O and stored in 70% ethanol. Specimens were dehydrated through a graded series of ethanol (70%, 80%, 95%, and 100%), cleared in xylene, infiltrated with 4 changes of paraffin, and embedded in fresh paraffin. Afterward, 3.5 μm thick sections were collected with a Reichert-Jung 820 microtome (Depew, NY), flattened on a warm water bath, mounted on glass slides, and stained with hematoxylin and eosin (H&E), as well as employing Masson’s trichrome (MT) stain to evaluate collagen components specifically. Additional process details are given in the file “A Guide to Perform Scaffold Characterization. pptx” within the repository).

### 2.5 Stromal collagen lamellae alignment and integrity

**FIGURE 2:**
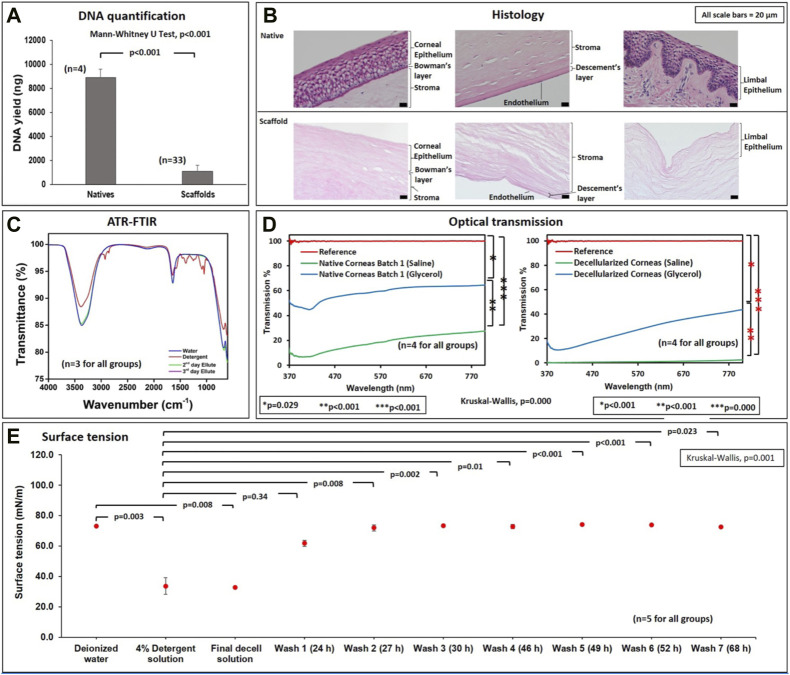
Biochemical, microscopic, and spectroscopic assessments of native and decellularized scaffolds. **(A)** A graphical representation of the relative DNA contents (ng DNA/μL of homogenized tissue) in native and acellular corneas. **(B)** Micrographs (from a ×20 objective) illustrate the effective removal and retention of extracellular matrix compartments in decellularized corneal scaffolds compared to native tissues. **(C)** FTIR assessments outline the effective removal of the detergent during the washout phase from the second and third day elutes. **(D)** Optical transmission studies verify the ability to retain viable degrees of ocular transparency in native and decellularized corneas, compared to the reference (glass slide), which roughly supports 100% light transmission. **(E)** Surface tension measurements of deionized water, 4% detergent, final decell solution (collected at the end of the seventh day of the decellularization process), and washouts obtained for three consecutive days (wash 1 through wash 7).

Based on the 20X microscopic images of collagen staining for corneal scaffolds (Figure 2), the characteristic fiber alignment and integrity of native and acellular segments were manually quantified. Twenty-two images for each group (native and decellularized) were randomly examined, and six areas were selected randomly selected in each image. These regions were used to examine morphological changes in the corneal stroma that result from decellularization. This approach was made by recording various regions of interest (ROIs) to separately gauge stromal fiber alignment and integrity, resulting in a score of either 0 or 1, whereby 0 represents the intact/characteristic alignment or integrity of the lamellae, whereas 1 represents altered/uncharacteristic lamellae alignment or integrity.

### 2.6 Attenuated total reflectance Fourier transform infrared spectroscopy

Attenuated total reflectance Fourier transform infrared spectroscopy (ATR-FTIR) spectra were obtained using a Spectrum Two FT-IR Spectrometer (Perkin Elmer, Waltham, MA, United States). All spectra were collected in the range of 4,000 to 600 cm^−1^ averaging 32 scans at a nominal resolution of 4 cm^−1^ for three randomly selected samples (*n* = 3). Spectra analyses were done using Spectrum software (Perkin Elmer, Waltham, MA, United States) on data from deionized water, zwitterionic biosurfactant, and second and third day washout solutions (elutes). Wavenumber (cm^−1^)-transmittance (%) data were obtained from Spectrum software and plotted with OriginPro 9.0.0 (64-bit) SR2 b87 (OriginLab Corporation, Northampton, MA, United States).

### 2.7 Surface tension

Surface tension data was acquired by a BP100 Bubble Pressure Tensiometer (Kruss Scientific, Matthews, United States). Data The data was collected and plotted using KRÜSS lab desktop software 3.2.2.3068 and Microsoft Excel. To collect such data, various elutes from the washout process were taken at different time intervals whenever the washing solutions were changed, as discussed in Section 2.2. After that, the surface tension of wash 1 (24 h), wash 2 (27 h), wash 3 (30 h), wash 4 (46 h), wash 5 (49 h), wash 6 (52 h), and wash 7 (68 h) solutions were measured. These measurements were compared with data measurements from deionized water, the zwitterionic decellularization agent (4% detergent solution), which were taken as controls, and the decellularization solution collected at the end of seventh day of the decellularization process (final decell solution).

### 2.8 Optical transmittance

Transmission spectra of the corneal scaffolds were obtained using a UV-Vis spectrophotometer connected to an optical microscope, Zeiss Axioscope (Zeiss Group, Oberkochen, Germany), through a fiber optic probe. The utilized spectrophotometer was USB 4000+ (Ocean Optics, Dunedin, FL, United States), which has an operation range of 200–1,100 nm, and the spectra were recorded using OceanView software. A glass slide was used as a reference for this experiment, and triplicate measurements of each sample were recorded and averaged. The spectra of the scaffolds, stored in saline, were initially measured. Then, upon placing the scaffolds in pure glycerol for 20 min, their surfaces were wiped with delicate task wipes, and their corresponding optical characteristics were recorded. The transmission spectra data were then averaged for each decellularized and native corneal sample placed in both saline and glycerol.

### 2.9 Corneal thickness

The thickness of each sample before, after decellularization, and after glycerol treatment was measured using a digital caliper (Insize, Loganville, GA, United States).

### 2.10 Statistical analysis

The Mann-Whitney U Test evaluated decellularization efficiency by comparing the DNA quantities in native corneas and decellularized scaffolds. The Kruskal–Wallis one-way analysis of variance (ANOVA) with the *post hoc* Dunn’s test was used to compare the results from the ocular transmission and surface tension studies. A *p*-value of less than 0.05 was considered statistically significant for all evaluations. All non-parametric statistical analyses were performed using SPSS (IBM Corp, Armonk, NY, United States). The characterization of variations in collagen fiber alignment and integrity are expressed as mean ± standard deviation.

## 3 Results

### 3.1 DNA quantification

DNA quantifications showed that the average yield for ≈ 25 mg tissue was approximately 1,100 ng in decellularized scaffolds (*n* = 33) and 8,800 ng in native tissues (*n* = 4), it was (Figure 2A). These results showed that nearly 90% of the native DNA was effectively removed from the corneal tissues through decellularization. Moreover, the Mann-Whitney U Test revealed that these differences in DNA contents were significantly different (*p* < 0.001).

### 3.2 Histological assessments

H&E staining in Figure 2B shows that this decellularization technique effectively removed nuclear and other cellular components from the endothelial, epithelial, stromal, and limbal regions of the extracted corneal tissues. Furthermore, the decellularized ECM appeared to retain an anatomical structure comparable to the original ECM.

### 3.3 Assessments of stromal collagen lamellae alignment and integrity

**FIGURE 3:**
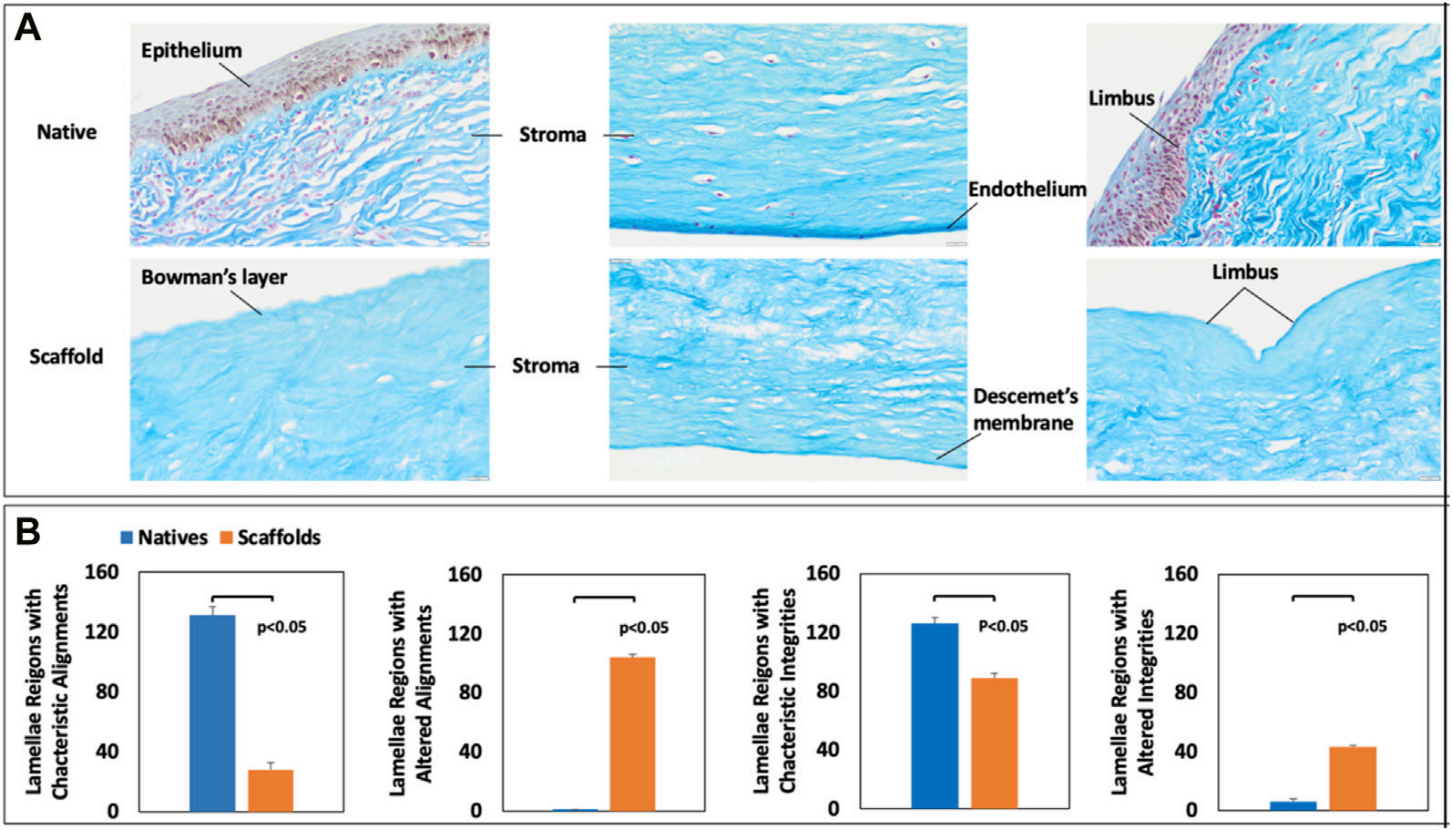
Histological assessment of native and decellularized scaffold collagen fiber alignment and integrity. MT staining of **(A)** native and decellularized corneal segments and manual assessments of **(A)** fiber alignments and **(B)** fiber integrities in native and decellularized scaffolds.

From Figure 3, the collagen fibers in the corneal stoma were stained blue, while the nuclei were stained dark pink. The alignment of the collagen fibers in the decellularized corneal was substantially different from the fibers present in the native tissues. Furthermore, we also observed significant alterations to the innate fiber integrity post-decellularization. Beyond these effects, MT staining also confirmed the effective removal of cellular components via decellularization. Meanwhile, this histological assessment provided evidence of the intactness of the Bowman’s layer, Descemet’s membrane, and limbus post-decellularization.

### 3.4 ATR-FTIR

ATR-FTIR spectroscopy was performed on several elutes to assess the effectiveness of the washout process. Spectra in Figure 2C were collected within the frequency band, 600 and 4,000 cm^−1^, to detect the molecular peaks corresponding to detergent-based components in the elutes using deionized water and pure detergent solution as references. No peaks corresponding to pure detergent were detected in the second day (wash 2) and third day (wash 3) elutes. Specifically, the spectra obtained from the elutes coincided with the spectra of deionized water, providing evidence of the effectual removal of the biosurfactant during the washout process.

### 3.5 Surface tension

The surface tension data is presented in Figure 2D. The surface tension of deionized water was, on average, 73.0 mN/m. This value was far greater than the mean surface tensions of the 4% detergent agent at the start and end of decellularization. In particular, the surface tension of the original biosurfactant was 33.6 mN/m, and the surface tension of the final decellularization solution (decell solution), collected after the 7-day corneal decellularization process, was 32.9 mN/m. Nevertheless, the surface tensions of washout solutions collected at different intervals were similar to the value for deionized water, which started from 61.7 mN/m for wash 1 (elute collected after the first day of washout process) and gradually increased to 72.6 mN/m for wash 7 (elute collected after the seventh day of washout process). The Kruskal–Wallis test also detected a significant difference (H = 38.609, *p* < 0.001) among the mean surface tension values recorded for all solutions of interest. Likewise, the *post hoc* Dunn’s pairwise comparisons revealed significant differences between the average surface tensions for the 4% detergent and deionized water and all washout solutions (*p* < 0.05 for all cases). Similar differences were observed between the final decell solution, deionized water, and all washout solutions (*p* < 0.05 for all cases). Nevertheless, this *ad-hoc* test was unable to detect a significant difference in the mean tensions of deionized water and all the washout solutions (*p* > 0.05 for all cases), as well as the mean tensions for the 4% detergent and the decell solution (*p* = 0.778).

### 3.6 Optical transmittance

The rates of light transmission through the native corneas and decellularized scaffolds stored in saline ranged between 7%–28% and 0%–3%, respectively (Figure 2C). Comparatively, after treatment with glycerol, transmission rates increased to 45%–65% for native corneas and 11%–45% for decellularized scaffolds. The Kruskal–Wallis test indicated a significant difference (H = 91.349, *p* = 0.000) between the ocular transmissions of natives and decellularized scaffolds. Moreover, the *post hoc* Dunn’s pairwise tests revealed significant differences between the following pairs: native corneas stored in saline and native corneas treated with glycerol (*p* < 0.001), decellularized corneas stored in saline and those in glycerol (*p* < 0.001), and native corneas and decellularized corneas which were both stored in saline (*p* = 0.006).

### 3.7 Corneal thickness

**FIGURE 4:**
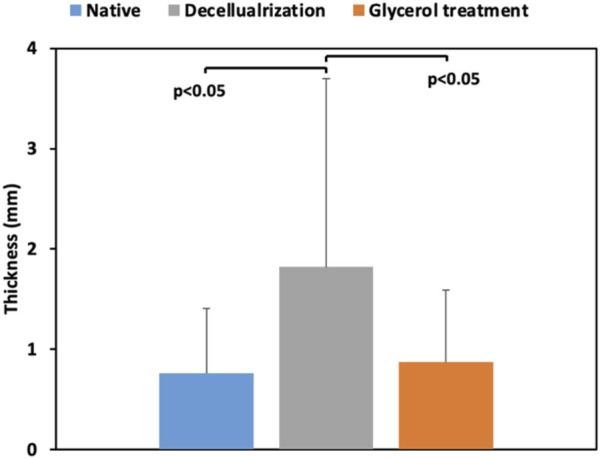
A comparison of the thicknesses of the native corneal segments (Native) with the scaffolds directly after decellularization (Decellularization) and post-treatment with glycerol (Glycerol treatment).

We measured the thickness of each sample before, after decellularization, and after glycerol treatment. This data is illustrated in Figure 4. The decellularization process substantially increased the corneal thickness. Our statistical analyses revealed significant variations between the average thickness of decellularized scaffolds, the native segments, and the acellular segments post-glycerol treatment. Moreover, glycerol appeared to support a reduced thickness, enabling the scaffolds to return to their original thickness.

## 4 Discussion and conclusion

It is challenging to balance the complete removal of all cellular components from a whole organ and the loss of integral ECM components through decellularization (Moffat et al., 2022; Wang Xe et al., 2023). As a result, researchers like Crapo et al. have proposed thresholds to delineate critical limits for acellular templates (Crapo et al., 2011). Our spectrophotometer-based DNA quantification measurements revealed an approximate 90% reduction in DNA content post-decellularization, which satisfy this criterion. Also, as anticipated, histological assessments allowed the visualization of nuclei and the ECM components (primarily collagen) in native sections. In comparison, such assessments highlight the effective removal of cellular compartments and retention of ECM components and confirm successful decellularization (Corridon et al., 2017; Garreta et al., 2017; Gilpin and Yang, 2017; Schmitt et al., 2017; Hillebrandt et al., 2019; Corridon, 2021; Polisetti et al., 2021; Wang et al., 2022a; Shakeel and Corridon, 2022b; Wang et al., 2022b; Corridon, 2022; Neishabouri et al., 2022; Pantic et al., 2022; Zhang et al., 2022; Corridon, 2023b; Khan et al., 2023).

Furthermore, for decades, infrared-based systems have offered a quantitative and qualitative analysis of biological samples for various applications (Corridon et al., 2006; Fadlelmoula et al., 2022; Cai et al., 2022; Corridon, 2023; Corridon et al., 2013; Hall et al., 2013; Kolb et al., 2018; Shaya et al., 2022; Corridon et al., 2021; Collett et al, 2017). For our purposes, this infrared-based approach provided evidence of the effective removal of appreciable levels of residual detergent and cellular/tissue debris, via the absence of spectral peaks in the frequency range 2,849 to 3075 cm^−1^ (Movasaghi et al., 2008) in the elute collected on the second and third day during the washout process (Figure 2C). Surface tension analyses further validated ATR FTIR findings (Figure 2E). Specifically, the gradual increase in surface tension from the original decellularizing agent (4% detergent solution) and decell solutions to the final elutes signify the steady and effectual removal of the detergent via the washout process. These observations are consistent with the fact that detergents substantially reduce the surface tension of water (Singh, 2006; Wang et al., 2012).

Finally, optical transmission tests showed a substantial variation in the rates of visible light transmitted through decellularized corneas compared to native corneas, which can be attributed to the experimental conditions. Corneal opacity in native tissues can be generated through the tissue extraction and storage processes, and this degree of opacity is heightened with decellularization. Overall, the presence of multiple scattering layers in biological tissues limits optical penetration depth and hence low transmission. The tissue components (including cells, membranes, collagen, fibrin, and elastin fibers) have refractive indices from 1.47 to 1.51, while the surrounding saline solution has a refractive index of nearly 1.33 (Costantini et al., 2019; Hamdy and Abdelazeem, 2020). This mismatch between the refractive indexes creates interferences from which light diffuses and scatters. Likewise, the substantial enhancements in corneal thickness, as wells as the alterations to collagen fiber alignment and integrity generated from decellularization, can provide additional hindrances to light propagation. By using a solvent with a higher refractive index, such as glycerol, with a refractive index of 1.47 (Bochert et al., 2005; Hedhly et al., 2022), index matching is achieved, reducing the optical inhomogeneity in the tissue samples. As shown in Figure 2D, the index-matching agent glycerol facilitated the reversal of ocular opacity (Figure 1) and enhanced the degree of light transmittance in these xenografts. Glycerol is a well-known dehydrating agent, with additional abilities to function as an antiprotease agent (Feilmeier et al., 2010). Thus, this compound aptly supported the recovery of corneal optical functional and innate characteristics, as previously noted in other studies (Li et al., 2012; Niu et al., 2019; Pantic et al., 2023d).

In conclusion, we present data for designing a simplified, inexpensive, scalable corneal xenograft platform. The platform can generate corneal scaffolds that are effectively immune to cellular components, comparable to native corneas in structure and transparency. The surfactant (detergent) employed is widely available. Besides, many eyeballs could be decellularized simultaneously in a scalable fashion. The strategies we applied in the whole eye decellularization process were efficient and affordable. While this process provides an appreciable degree of optical transmittance, it is significantly lower than the degree of transmittance that several artificial substitutes that can support 70%–90% (Tidu et al., 2020). Nevertheless, examining the ability to decellularize the organ directly reduces the need for specialized personnel to isolate the cornea upfront and reduce the potential time for tissue degradation, as observed in another study from our group that examined the decellularization of the extracted cornea (Pantic et al., 2023d). The agitation process also expedited the scaffold creation as the physical method of combining chemical agents. Compared to other combinational decellularization approaches, ours are simpler and more efficient. Specifically, to recover the ocular transparency from corneal replacements, scaffolds can be immersed in glycerol and exerted with shaking (agitation), which enhances the contact and interactions between glycerol and specimens resulting in high efficiency. Even though we displayed the data from surface tension tests revealed that the washing-out procedure has a high potential for removing detergent residuals, more work is required to examine the effectiveness of this process. Therefore, future studies will focus on biocompatibility and toxicity tests for further validation and scaffold recellularization.

Furthermore, collagen-based materials are the most common, as collagen is the most abundant component of the corneal stroma (Fernández-Pérez and Ahearne, 2020). Maintenance of these components will help provide a suitable environment for future recellularization via direct cell seeding and bioreactor approaches outlined in the literature (Fernández-Pérez and Ahearne, 2020) and future integration into the ocular surface and accommodating the presence of other tissues such as conjunctiva, buccal mucous membrane, or periosteum. While this is early development, future studies must evaluate essential elements like suture retention, neovascularization, graft stability, and tissue dynamics to support long-term transplantation. This high-throughput strategy can produce several native ovine xenografts and acellular corneal scaffolds by repurposing slaughterhouse waste to support circular economic practices. Thus, such a sustainable source, these efforts can extend current tissue engineering applications and lay a new foundation for biomimetic corneal graft banking.

## 5 Database description

The procedures of cornea sectioning and analysis and the results for DNA quantification, histology, FTIR, and ocular transmission are presented in our repository in the file called Slaughterhouse_waste_ocular_repruposing_usage_notes.csv. This file outlines specific details on the ways the data was handled. Our research group and others in the field have formerly used similar assays to analyze the decellularization efficacy and scaffold viability, and the related citations have been outlined. The repository is publicly available under a Creative Commons CCO license to support further analysis and publication, with the requirement to cite this article and the dataset.

## Data Availability

The datasets presented in this study can be found in online repositories. The names of the repository/repositories and accession number(s) can be found below: https://figshare.com/articles/online_resource/Detailed_descriptions_for_high‐throughput_repurposing_of_discarded_abattoir_ovine_eyes_to_generate_corneal_grafts_and_aid_circular_economic_practices_in_the_United_Arab_Emirates/21270399.

## References

[B1] Ahearne M. (2020). “Chapter 5 - corneal extracellular matrix decellularization,” in Methods in cell biology. Editors Caballero D. Kundu S. C. Reis R. L. (Academic Press), 157, 81–95. 10.1016/bs.mcb.2019.10.013 32334721

[B2] Ahearne M. Fernández-Pérez J. Masterton S. Madden P. W. Bhattacharjee P. (2020). Designing scaffolds for corneal regeneration. Adv. Funct. Mater. 30 (44), 1908996. 10.1002/adfm.201908996

[B3] Bernard A. He Z. Forest F. Gauthier A-S. Peoc'h M. Dumollard J-M. (2015). Femtosecond laser cutting of multiple thin corneal stromal lamellae for endothelial bioengineering. Cornea 34 (2), 218–224. 10.1097/ico.0000000000000306 25474234

[B4] Bochert R. Zhivov A. Kraak R. Stave J. Guthoff R. (2005). Contribution to comprehension of image formation in confocal microscopy of cornea with Rostock cornea module. Br. J. Ophthalmol. 89, 1351–1355. 10.1136/bjo.2004.063743 16170131 PMC1772867

[B59] Cai N. Lai A. C. Liao K. Corridon P. R. Graves D. J. Chan V. (2022). Recent Advances in Fluorescence Recovery after Photobleaching for Decoupling Transport and Kinetics of Biomacromolecules in Cellular Physiology. Polymers (Basel) 14 (9). 10.3390/polym14091913 PMC9105003 35567083

[B5] Corridon P. Ascázubi R. Krest C. Wilke I. (Editors) (2006). Time-domain terahertz spectroscopy of artificial skin (ProcSPIE).

[B6] Corridon P. (2023). Still finding ways to augment the existing management of acute and chronic kidney diseases with targeted gene and cell therapies: Opportunities and hurdles. Preprints 10, 1143028. 10.3389/fmed.2023.1143028 PMC10028138 36960337

[B7] Corridon P. R. (2023). Capturing effects of blood flow on the transplanted decellularized nephron with intravital microscopy. Sci. Rep. 13 (1), 5289. 10.1038/s41598-023-31747-w 37002341 PMC10066218

[B60] Corridon P. R. (2023). Enhancing the expression of a key mitochondrial enzyme at the inception of ischemia-reperfusion injury can boost recovery and halt the progression of acute kidney injury. Front Physiol. 14 (4), 1024238. 36846323 10.3389/fphys.2023.1024238 PMC9945300

[B8] Corridon P. R. (2021). *In vitro* investigation of the impact of pulsatile blood flow on the vascular architecture of decellularized porcine kidneys. Sci. Rep. 11 (1), 16965. 10.1038/s41598-021-95924-5 34417499 PMC8379263

[B9] Corridon P. R. (2022). Intravital microscopy datasets examining key nephron segments of transplanted decellularized kidneys. Sci. Data 9 (1), 561. 10.1038/s41597-022-01685-9 36088356 PMC9464233

[B10] Corridon P. R. Ko I. K. Yoo J. J. Atala A. (2017). Bioartificial kidneys. Curr. Stem Cell Rep. 3 (2), 68–76. 10.1007/s40778-017-0079-3 33005562 PMC7526744

[B11] Corridon P. R. Wang X. Shakeel A. Chan V. (2022). Digital technologies: Advancing individualized treatments through gene and cell therapies, pharmacogenetics, and disease detection and diagnostics. Biomedicines 10 (10), 2445. 10.3390/biomedicines10102445 36289707 PMC9599083

[B61] Corridon P. R. Rhodes G. J. Leonard E. C. Basile D. P. Gattone V. H. Bacallao R. L. (2013). A method to facilitate and monitor expression of exogenous genes in the rat kidney using plasmid and viral vectors. Am J Physiol Renal Physiol. 304 (9), F1217–F1229. 23467422 10.1152/ajprenal.00070.2013 PMC3651629

[B65] Corridon P. R. Karam S. H. Khraibi A. A. Khan A. A. Alhashmi M. A. (2021). Intravital imaging of real-time endogenous actin dysregulation in proximal and distal tubules at the onset of severe ischemia-reperfusion injury. Sci. Rep. 11 (1), 8280. 33859322 10.1038/s41598-021-87807-6 PMC8050301

[B66] Collett J. A. Corridon P. R. Mehrotra P. Kolb A. L. Rhode G. J. Miller C. A (2017). Hydrodynamic Isotonic Fluid Delivery Ameliorates Moderate-to-Severe Ischemia-Reperfusion Injury in Rat Kidneys. J. Am. Soc. Nephrol. 28 (7), 2081–92. 28122967 10.1681/ASN.2016040404 PMC5491274

[B12] Costantini I. Cicchi R. Silvestri L. Vanzi F. Pavone F. S. (2019). *In-vivo* and *ex-vivo* optical clearing methods for biological tissues: Review. Biomed. Opt. Express 10 (10), 5251–5267. 10.1364/boe.10.005251 31646045 PMC6788593

[B13] Crapo P. M. Gilbert T. W. Badylak S. F. (2011). An overview of tissue and whole organ decellularization processes. Biomaterials 32 (12), 3233–3243. 10.1016/j.biomaterials.2011.01.057 21296410 PMC3084613

[B14] Davidovic L. M. Cumic J. Dugalic S. Vicentic S. Sevarac Z. Petroianu G. (2022). Gray-Level Co-occurrence matrix analysis for the detection of discrete, ethanol-induced, structural changes in cell nuclei: An artificial intelligence approach. Microsc. Microanal. 28 (1), 265–271. 10.1017/s1431927621013878 34937605

[B15] Fadlelmoula A. Pinho D. Carvalho V. H. Catarino S. O. Minas G. (2022). Fourier transform infrared (FTIR) spectroscopy to analyse human blood over the last 20 Years: A review towards lab-on-a-chip devices. Micromachines (Basel) 13 (2), 187. 10.3390/mi13020187 35208311 PMC8879834

[B16] Feilmeier M. R. Tabin G. C. Williams L. Oliva M. (2010). The use of glycerol-preserved corneas in the developing world. Middle East Afr. J. Ophthalmol. 17 (1), 38–43. 10.4103/0974-9233.61215 20543935 PMC2880372

[B17] Fernández-Pérez J. Ahearne M. (2020). Decellularization and recellularization of cornea: Progress towards a donor alternative. Methods 171, 86–96. 10.1016/j.ymeth.2019.05.009 31128238

[B18] Fischer K. Schnieke A. (2022). Xenotransplantation becoming reality. Transgenic Res. 31 (3), 391–398. 10.1007/s11248-022-00306-w 35545691 PMC9135885

[B19] Garreta E. Oria R. Tarantino C. Pla-Roca M. Prado P. Fernández-Avilés F. (2017). Tissue engineering by decellularization and 3D bioprinting. Mater. Today 20 (4), 166–178. 10.1016/j.mattod.2016.12.005

[B20] Gerasimidis K. Bertz M. Quince C. Brunner K. Bruce A. Combet E. (2016). The effect of DNA extraction methodology on gut microbiota research applications. BMC Res. Notes 9 (1), 365. 10.1186/s13104-016-2171-7 27456340 PMC4960752

[B21] Gilpin A. Yang Y. (2017). Decellularization strategies for regenerative medicine: From processing techniques to applications. Biomed. Res. Int. 2017, 1–13. 10.1155/2017/9831534 PMC5429943 28540307

[B22] Hamdy O. Abdelazeem R. (2020). Toward better medical diagnosis: Tissue optical clearing. J. Public Health Int. 2, 13–21. 10.14302/issn.2641-4538.jphi-19-3132

[B62] Hall A. M. Rhodes G. J. Sandoval R. M. Corridon P. R. Molitoris B. A. (2013). *In vivo* multiphoton imaging of mitochondrial structure and function during acute kidney injury. Kidney Int. 83 (1), 72–83. 22992467 10.1038/ki.2012.328 PMC4136483

[B23] Hedhly M. Wang Y. Zeng S. Ouerghi F. Zhou J. Humbert G. (2022). Highly sensitive plasmonic waveguide biosensor based on phase singularity-enhanced goos–hänchen shift. Biosensors 12 (7), 457. 10.3390/bios12070457 35884260 PMC9312834

[B24] Hillebrandt K. H. Everwien H. Haep N. Keshi E. Pratschke J. Sauer I. M. (2019). Strategies based on organ decellularization and recellularization. Transpl. Int. official J. Eur. Soc. Organ Transplant. 32 (6), 571–585. 10.1111/tri.13462 31099920

[B25] Isidan A. Liu S. Chen A. M. Zhang W. Li P. Smith L. J. (2021). Comparison of porcine corneal decellularization methods and importance of preserving corneal limbus through decellularization. PLoS One 16 (3), e0243682. 10.1371/journal.pone.0243682 33667231 PMC7935270

[B63] Kolb A. L. Corridon P. R. Zhang S. Xu W. Witzmann F. A. Collett J. A. (2018). Exogenous Gene Transmission of Isocitrate Dehydrogenase 2 Mimics Ischemic Preconditioning Protection. J Am Soc Nephrol. 29 (4), 1154–1164. 29371417 10.1681/ASN.2017060675 PMC5875948

[B26] Khan R. L. Khraibi A. A. Dumée L. F. Corridon P. R. (2023). From waste to wealth: Repurposing slaughterhouse waste for xenotransplantation. Front. Bioeng. Biotechnol. 11, 1091554. 10.3389/fbioe.2023.1091554 36815880 PMC9935833

[B27] Kong B. Mi S. (2016). Electrospun scaffolds for corneal tissue engineering: A review. Mater. (Basel) 9 (8), 614. 10.3390/ma9080614 PMC5509008 28773745

[B28] Kumar K. P. Tejashree A. (2022). Genotyping of Candida albicans and comparison of its antifungal resistance pattern in the south Indian region. J. Pure Appl. Microbiol. 16, 2123–2130. 10.22207/jpam.16.3.69

[B29] Li J. Shi S. Zhang X. Ni S. Wang Y. Curcio C. A. (2012). Comparison of different methods of glycerol preservation for deep anterior lamellar keratoplasty eligible corneas. Investigative Ophthalmol. Vis. Sci. 53 (9), 5675–5685. 10.1167/iovs.12-9936 22836770

[B30] Moffat D. Ye K. Jin S. (2022). Decellularization for the retention of tissue niches. J. Tissue Eng. 13, 204173142211011. 10.1177/20417314221101151 PMC9128068 35620656

[B31] Mousa M. Vurivi H. Kannout H. Uddin M. Alkaabi N. Mahboub B. (2021). Genome-wide association study of hospitalized COVID-19 patients in the United Arab Emirates. EBioMedicine 74, 103695. 10.1016/j.ebiom.2021.103695 34775353 PMC8587122

[B32] Movasaghi Z. Rehman S. ur Rehman D. I. (2008). Fourier transform infrared (FTIR) spectroscopy of biological tissues. Appl. Spectrosc. Rev. 43 (2), 134–179. 10.1080/05704920701829043

[B33] Neishabouri A. Soltani Khaboushan A. Daghigh F. Kajbafzadeh A. M. Majidi Zolbin M. (2022). Decellularization in tissue engineering and regenerative medicine: Evaluation, modification, and application methods. Front. Bioeng. Biotechnol. 10, 805299. 10.3389/fbioe.2022.805299 35547166 PMC9081537

[B34] Niu G. Zhou Q. Huang X. Wang S. Zhang J. Zhang Y. (2019). Individualized penetrating keratoplasty using edge-trimmed glycerol-preserved donor corneas for perforated corneal ulcers. BMC Ophthalmol. 19 (1), 85. 10.1186/s12886-019-1091-4 30940116 PMC6444435

[B35] Panek M. Čipčić Paljetak H. Barešić A. Perić M. Matijašić M. Lojkić I. (2018). Methodology challenges in studying human gut microbiota – effects of collection, storage, DNA extraction and next generation sequencing technologies. Sci. Rep. 8 (1), 5143. 10.1038/s41598-018-23296-4 29572539 PMC5865204

[B36] Pantic I. Cumic J. Dugalic S. Petroianu G. A. Corridon P. R. (2023). Gray level co-occurrence matrix and wavelet analyses reveal discrete changes in proximal tubule cell nuclei after mild acute kidney injury. Sci. Rep. 13 (1), 4025. 10.1038/s41598-023-31205-7 36899130 PMC10006226

[B37] Pantic I. Cumic J. Valjarevic S. Shakeel A. Wang X. (2023). Computational approaches for evaluating morphological changes in the corneal stroma associated with decellularization. 16 January 2023, PREPRINT (Version 1) available at Research Square. 10.21203/rs.3.rs-2480023/v1 PMC10250676 37304146

[B38] Pantic I. Paunovic J. Cumic J. Valjarevic S. Petroianu G. A. Corridon P. R. (2023). Artificial neural networks in contemporary toxicology research. Chemico-Biological Interact. 369, 110269. 10.1016/j.cbi.2022.110269 36402212

[B39] Pantic I. Valjarevic S. Cumic J. Paunkovic I. Terzic T. Corridon P. R. (2023). Gray level Co-occurrence matrix, fractal and wavelet analyses of discrete changes in cell nuclear structure following osmotic stress: Focus on machine learning methods. Fractal Fract. 7 (3), 272. 10.3390/fractalfract7030272

[B40] Pantic I. V. Shakeel A. Petroianu G. A. Corridon P. R. (2022). Analysis of vascular architecture and parenchymal damage generated by reduced blood perfusion in decellularized porcine kidneys using a gray level Co-occurrence matrix. Front. Cardiovasc. Med. 9, 797283. 10.3389/fcvm.2022.797283 35360034 PMC8963813

[B41] Polisetti N. Schmid A. Schlötzer-Schrehardt U. Maier P. Lang S. J. Steinberg T. (2021). A decellularized human corneal scaffold for anterior corneal surface reconstruction. Sci. Rep. 11 (1), 2992. 10.1038/s41598-021-82678-3 33542377 PMC7862698

[B42] Schmitt A. Csiki R. Tron A. Saldamli B. Tubel J. Florian K. (2017). Optimized protocol for whole organ decellularization. Eur. J. Med. Res. 22 (1), 31. 10.1186/s40001-017-0272-y 28886732 PMC5591503

[B43] Shafiq M. A. Gemeinhart R. A. Yue B. Y. Djalilian A. R. (2012). Decellularized human cornea for reconstructing the corneal epithelium and anterior stroma. Tissue Eng. Part C Methods 18 (5), 340–348. 10.1089/ten.tec.2011.0072 22082039 PMC3338110

[B44] Shakeel A. Corridon P. R. (2022). Mitigating challenges and expanding the future of vascular tissue engineering-are we there yet? Front. Physiol. 13, 1079421. 10.3389/fphys.2022.1079421 36685187 PMC9846051

[B45] Shakeel A. Corridon P. R. (2022). Mitigating challenges and expanding the future of vascular tissue engineering–are we there yet? Available at SSRN 4039050. 2022. 10.3389/fphys.2022.1079421 PMC9846051 36685187

[B64] Shaya J. Corridon P. R. Al-Omari B. Aoudi A. Shunnar A. Mohideen M. I. H. (2022). Design, photophysical properties, and applications of fluorene-based fluorophores in two-photon fluorescence bioimaging: A review. J. Photochem. Photobiol. C: Photochem review 52, 100529.

[B46] Singh M. (2006). Measurements of surface tension and viscosity of liquids with survismeter - a green chemistry instrumental approach. Chemistry 15, 426–430.

[B47] Song Y. W. Pan Z. Q. (2019). Reducing porcine corneal graft rejection, with an emphasis on porcine endogenous retrovirus transmission safety: A review. Int. J. Ophthalmol. 12 (2), 324–332. 10.18240/ijo.2019.02.21 30809491 PMC6376240

[B48] Tidu A. Schanne-Klein M-C. Borderie V. M. (2020). Development, structure, and bioengineering of the human corneal stroma: A review of collagen-based implants. Exp. Eye Res. 200, 108256. 10.1016/j.exer.2020.108256 32971095

[B49] Wang X. Chan V. Corridon P. R. (2022). Acellular tissue-engineered vascular grafts from polymers: Methods, achievements, characterization, and challenges. Polym. (Basel) 14 (22), 4825. 10.3390/polym14224825 PMC9695055 36432950

[B50] Wang X. Chan V. Corridon P. R. (2022). Decellularized blood vessel development: Current state-of-the-art and future directions. Front. Bioeng. Biotechnol. 10, 951644. 10.3389/fbioe.2022.951644 36003539 PMC9394443

[B51] Wang X. Shakeel A. Salih A. E. Vurivi H. Daoud S. Desidery L. (2022). High-throughput abattoir ovine eyes repurposed for corneal xenografting and circular economic practices. Figshare. 10.6084/m9.figshare.21270399

[B52] Wang Xe R. Abdukadir A. M. Ali Z. M. Corridon P. (2023). A proposed model of xeno-keratoplasty using 3D printing and decellularization. Preprintsorg, 2023030522. 10.20944/preprints2023.030522.v1 PMC10548234 37799970

[B53] Wang Z. Shen Y. Ma J. Haapasalo M. (2012). The effect of detergents on the antibacterial activity of disinfecting solutions in dentin. J. Endod. 38 (7), 948–953. 10.1016/j.joen.2012.03.007 22703659

[B54] Wilson S. L. Sidney L. E. Dunphy S. E. Dua H. S. Hopkinson A. (2016). Corneal decellularization: A method of recycling unsuitable donor tissue for clinical translation? Curr. Eye Res. 41 (6), 769–782. 10.3109/02713683.2015.1062114 26397030 PMC4926783

[B55] Wilson S. L. Sidney L. E. Dunphy S. E. Rose J. B. Hopkinson A. (2013). Keeping an eye on decellularized corneas: A review of methods, characterization and applications. J. Funct. Biomater. 4 (3), 114–161. 10.3390/jfb4030114 24956084 PMC4030906

[B56] Xie L. Ouyang C. Ji J. Wu J. Dong X. Hou C. (2021). Construction of bioengineered corneal stromal implants using an allogeneic cornea-derived matrix. Mater. Sci. Eng. C 120, 111673. 10.1016/j.msec.2020.111673 33545838

[B57] Yoon C. H. Choi H. J. Kim M. K. (2021). Corneal xenotransplantation: Where are we standing? Prog. Retin Eye Res. 80, 100876. 10.1016/j.preteyeres.2020.100876 32755676 PMC7396149

[B58] Zhang X. Chen X. Hong H. Hu R. Liu J. Liu C. (2022). Decellularized extracellular matrix scaffolds: Recent trends and emerging strategies in tissue engineering. Bioact. Mater. 10, 15–31. 10.1016/j.bioactmat.2021.09.014 34901526 PMC8637010

